# A Mechanistic Review on How Berberine Use Combats Diabetes and Related Complications: Molecular, Cellular, and Metabolic Effects

**DOI:** 10.3390/ph17010007

**Published:** 2023-12-20

**Authors:** Vahid Reza Askari, Kimia Khosravi, Vafa Baradaran Rahimi, Stefania Garzoli

**Affiliations:** 1 Applied Biomedical Research Center, Mashhad University of Medical Sciences, Mashhad 9177948564, Iran; vahidrezaaskary@gmail.com; 2 International UNESCO Center for Health-Related Basic Sciences and Human Nutrition, Mashhad University of Medical Sciences, Mashhad 9177948564, Iran; 3 School of Pharmacy, Mashhad University of Medical Sciences, Mashhad 1696700, Iran; khosravikimia232@gmail.com; 4 Department of Cardiovascular Diseases, Faculty of Medicine, Mashhad University of Medical Sciences, Mashhad 1696700, Iran; vafa_br@yahoo.com; 5 Department of Chemistry and Technologies of Drug, Sapienza University, P. le Aldo Moro, 5, 00185 Rome, Italy

**Keywords:** berberine, type 2 diabetes, streptozotocin, insulin, inflammation, oxidative stress, peroxisome proliferator-activated receptor-γ, glucose transporter-4

## Abstract

Berberine (BBR) is an isoquinoline alkaloid that can be extracted from herbs such as Coptis, Phellodendron, and Berberis. BBR has been widely used as a folk medicine to treat various disorders. It is a multi-target drug with multiple mechanisms. Studies have shown that it has antioxidant and anti-inflammatory properties and can also adjust intestinal microbial flora. This review focused on the promising antidiabetic effects of BBR in several cellular, animal, and clinical studies. Based on previous research, BBR significantly reduced levels of fasting blood glucose, hemoglobin A1C, inflammatory cytokines, and oxidative stress markers. Furthermore, BBR stimulated insulin secretion and improved insulin resistance through different pathways, including up-regulation of protein expression of proliferator-activated receptor (PPAR)-γ, glucose transporter (GLUT) 4, PI3K/AKT, and AMP-activated protein kinase (AMPK) activation. Interestingly, it was demonstrated that BBR has protective effects against diabetes complications, such as diabetic-induced hepatic damage, cardiovascular disorders, nephropathy, and neuropathy. Furthermore, multiple clinical trial studies have emphasized the ameliorative effects of BBR in type 2 diabetic patients.

## 1. Introduction

Type 2 diabetes mellitus (T2DM), one of the most widespread persistent diseases, is characterized by hyperglycemia. Its incidence rate is constantly increasing. In 2021, 529 million people suffered from diabetes. It is predicted that this number will reach 1.3 billion by 2050 [1]. Prescription drugs and insulin supplementation represent the current primary treatment of T2DM; however, some adverse effects have been noted, including liver difficulties and lactic acidosis [2]. Consequently, an ongoing search for alternative medicines and herbal remedies for T2DM with high efficacy and low toxicity is essential.

Berberine (BBR) is an isoquinoline alkaloid compound that can be isolated from Coptis, Phellodendron, and Berberis. BBR has been used for many years as a folk medicine in China in treating diarrhea and diabetes [3]. It is also known for its anticancer, anti-inflammatory, and antibacterial effects [4]. BBR also has an antidiabetic impact similar to metformin [5]. In addition to T2D, BBR has a beneficial effect on the complications of diabetes, including renal damage [6], neuropathy [7], retinopathy [8], cognitive problems [9], cardiovascular complications [10], and endothelial dysfunction [11], through various mechanisms. Furthermore, many clinical studies have confirmed the beneficial effects of BBR in diabetic patients. Therefore, BBR can be used as a treatment in diabetic patients. This review aims to provide updated and comprehensive information on the use of BBR in diabetes and its cellular and molecular mechanisms to support its therapeutic potential and to find insights for future research.

## 2. Search Strategy and Study Selection

A search was conducted on the PubMed, Scopus, Web of Science, and Science Direct databases, starting in March 2023. The publications included in this study were identified by using the following keywords: berberine, diabetes, inflammation, hypertension, atherosclerosis, nephropathy, neuropathy, retinopathy, and hepatic damage. All in vitro, in vivo, and clinical trials were included. There were no restrictions on publication language or publication date. We evaluated 367 works in the literature. Articles that did not meet the exclusion criteria were excluded. The final number of articles that we used in this article was 116.

## 3. Results and Discussion

### 3.1. In Vitro Models of Diabetes Mellitus (DM)

One study investigated the effects of short-term treatment with BBR on 3T3-L1 adipocytes and L6 myoblasts. BBR inhibited triglyceride accumulation in fully differentiated and undifferentiated adipocytes. In addition, it reduced adipogenic gene expression and levels of most lipogenic transcripts (including the Fas receptor, also known as APO-1 or CD95), adipocyte determination and differentiation–dependent factor 1/sterol regulatory element–binding protein 1c (ADD1/SREBP1c), peroxisome proliferator-activated receptors (PPARs), CCAAT/enhancer binding proteins (C/EBPs), 11beta-hydroxysteroid dehydrogenase 1 (11β-HSD1), and adipocyte protein 2 (aP2). BBR increased the phosphorylation of AMP-activated protein kinase (AMPK) and ACC (a major substrate of AMPK) in both adipocytes and myoblasts. Furthermore, it also increased GLUT4 translocation in myoblasts [12].

Another laboratory investigation was conducted on neonatal rat cardiomyocytes exposed to hypoxia/reoxygenation with elevated glucose levels. BBR, at a concentration of 50 µM, decreased hypoxia/reoxygenation-promoted myocardial cell death, increased the Bcl-2/Bax ratio, decreased caspase-3 expression, activated phosphoinositide 3-kinase (PI3K)–Akt, and amplified AMP-activated protein kinase (AMPK) and endothelial nitric oxide synthase (eNOS) phosphorylation. The fact that prior treatment with either the PI3K/Akt inhibitor wortmannin or the AMPK inhibitor compound C decreased the antiapoptotic effect of BBR supported the mechanisms of BBR [13]. In the same in vitro study, BBR and metformin, either alone or in combination, were tested on a high-glucose-induced hepatocellular carcinoma (HepG2) cell line in order to evaluate the effects of both on lipid levels [14].

HepG2 cells were treated with glucose (33 mM) for 24 h after being pretreated with BBR and metformin. Concentrations of 20 and 40 μM of BBR could reduce the total lipid content and triglycerides in the treated HepG2 cells. Synergistic effects in reducing total lipid contents and triglyceride levels in HepG2 cells were obtained following the simultaneous administration of metformin and BBR at ratios of 2:10, 1:10, 0.5:10, and 0.25%. Furthermore, the combination of metformin and BBR at the lowest concentrations (0.25 and 5 μM) also showed a synergistic effect and reduced the expression of FAS and SREBP-1c [14].

In another study, a high-glucose-induced H9C2 cell line mimicked type 2 diabetes-induced cardiomyocyte hypertrophy. The protective effect of BBR and its mechanism were investigated in this model. Cardiomyocyte hypertrophy is related to impaired mitochondrial function. Thus, BBR significantly reduced H9C2 cell line hypertrophy by improving mitochondrial function (correcting the fusion and fission imbalance in mitochondrial dynamics). Furthermore, BBR promoted mitogenesis and destroyed damaged mitochondria through mitophagy. It also restored autophagic flux in damaged cardiomyocytes by activating the AMPK signaling pathway [15]. All of the in vitro studies of BBR are summarized in Table 1.

### 3.2. Animal Models of Diabetes Mellitus (DM)

The promising antidiabetic effects of BBR have been reported in several animal models of DM, including streptozotocin (STZ) and alloxan-induced DM (Table 2).

#### 3.2.1. Protective Effects of Berberine against *db/db* and STZ-Induced DM

In STZ-induced diabetic rats, BBR exhibited antihyperglycemic, antioxidant, anti-inflammatory, and antiapoptotic activities. For example, in one study, a BBR chloride treatment (50 mg/kg/day) was administered orally to diabetic rats for forty-five days. When administered orally, BBR chloride significantly reduced blood glucose levels and HbAlc, while it increased plasma insulin, hemoglobin, and body weight. In addition, the pancreatic levels of superoxide dismutase (SOD), catalase (CAT), glutathione peroxidase (GPX), reduced glutathione (GSH), vitamin E, and vitamin C increased, while those of lipid peroxidation markers, i.e., lipid hydroperoxides (LOOHs) and 2-thiobarbituric acid reactive substances (TBARSs), decreased. Furthermore, in the BBR chloride-treated group, a significant decrease in pro-inflammatory mediators, including tumor necrosis factor (TNF), nuclear factor-kappa B (NF-κB), phospho-NF-κB-p65, cyclooxygenase-2 (COX-2), and inducible nitric oxide synthase (iNOS), as well as pro-apoptotic proteins, such as caspase-8, t-Bid, Bax, cytochrome-c, and cleaved caspase-3, was observed, while there was an increase in the anti-apoptotic protein Bcl-2, interleukin 10 (IL-10) as an anti-inflammatory mediator, and glucose transporter-2 (GLUT-2) [16].

In another study, rats were fed high-fat BBR laboratory food at dosages of 187.5 and 562.5 mg/kg for four weeks. After 4 weeks of BBR treatment, the results showed that oral glucose tolerance improved in the BBR-treated groups. In contrast, fasting blood glucose (FBG) (7.4 ± 1.5 or 7.3 ± 1.3 vs. 9.3 ± 1.3 mM), triglyceride (TG) (0.61 ± 0.22 or 0.63 ± 0.17 vs. 1.8 ± 0.7 mM), total cholesterol (TC) (1.8 ± 0.3 or 1.9 ± 0.3 vs. 2.2 ± 0.2 mM), free fatty acid (FFA) (456 ± 93 or 460 ± 72 vs. 550 ± 113 μM), and apolipoprotein B levels (0.37 ± 0.02 or 0.42 ± 0.05 vs. 0.46 ± 0.04 g/L) were reduced at BBR doses of 187.5 and 562.5 mg/kg/d, respectively, compared to the control group. In addition, the HDL (1.5 ± 0.3 or 1.4 ± 0.3 vs. 1.1 ± 0.1 g/L) and apolipoprotein AI (0.80 ± 0.08 or 0.87 ± 0.08 vs. 0.71 ± 0.06 g/L) levels were significantly increased [17].

It was shown that BBR reduced fasting plasma glucose (FPG); fasting insulin (FINS); the expression of TLR4, TNF-α, IL-1β and IL-6; pathological damage; and macrophage (MΦ) infiltration in pancreatic islets of diabetic mice. It also regulated probiotics in the intestinal tracts of diabetic mice. Furthermore, BBR blocked the nuclear translocation of NF-κB in THP-1-derived MΦs. Therefore, BBR plays a crucial role in regulating the gut microbiota and inhibiting the TLR4-NF-κB signaling pathway, and, through these mechanisms, it can suppress inflammation and alleviate symptoms related to diabetes [18]. It should be mentioned that the anti-inflammatory mechanism of BBR is to inhibit the binding between Toll-like receptor 4 (TLR4) and MyD88 and disturb the TLR4/MyD88/NF-κB signaling pathway [33].

In a previous study [19], BBR (156 mg/kg per day) showed a protective effect in STZ-induced diabetic rats by inhibiting hepatic gluconeogenesis. BBR reduced fasting plasma insulin, insulin resistance estimated by homeostasis model assessment (HOMA-IR), hyperlipidemia, and up-regulated protein expression of liver kinase B1 (LKB1), AMPK, and phosphorylated AMPK (p-AMPK). It also down-regulated the protein expression of key gluconeogenic enzymes (such as phosphoenolpyruvate carboxykinase and glucose-6phosphatase). Another mechanism of action of BBR was its impact on the target of rapamycin (TOR) complex-2 (TORC2) protein. The phosphorylated form of TORC2 (p-TORC2) was localized in the cytoplasm and prevented gluconeogenesis. Thus, BBR treatment increased p-TORC2 levels and inhibited TORC2 nuclear translocation in liver tissues. Furthermore, LKB1 has been shown to act as an upstream regulator of AMPK and has a role in gluconeogenesis. AMPK phosphorylation activates phosphorylation of the cAMP-response element binding protein (CREB)-regulated transcription co-activator TORC2, which results in the containment of TORC2 nuclear translocation. Thus, gluconeogenesis was inhibited [19].

Another study demonstrated that BBR (5 mg/kg/day) reduced blood glucose levels; improved insulin activity, glucose tolerance, and glucose metabolism; and decreased hepatic gluconeogenesis in the livers of *ob/ob* and STZ-induced diabetic mice. In addition, it decreased glucagon-induced glucose production and gluconeogenic gene expression in hepatocytes, apparently through cAMP reduction, and also suppressed glucagon-induced CREB phosphorylation [23].

Another study administered BBR intraperitoneally to *db/db* mice (5 mg/kg/d). The results showed that the body weights of the BBR-treated mice were significantly reduced even though the food intake did not change. BBR reduces fat mass primarily by decreasing the size of fat cells rather than their number. It also caused a significant improvement in glucose tolerance [12].

It was illustrated that BBR treatment (100 mg/kg/d for two weeks) reduced FBG to lower levels than in the control group. In BBR-treated mice, 15 min after glucose injection, the peak blood glucose appeared, while in vehicle mice and rosiglitazone-treated mice, the peaks appeared at 120 and 60 min, respectively, suggesting that BBR increased the sensitivity to insulin in *db/db* mice [24].

In another study, BBR reduced FBG and FINS and increased insulin receptor mRNA and PKC expression in STZ-induced T2DM rats and KK-Ay mice, but in animal models of type 1 diabetes (NOD/LtJ mice), BBR (100 mg/kg/day for three weeks) did not show hypoglycemic effects. Lack of insulin is the reason BBR did not protect NOD/LtJ mice from diabetes [20]. Another study was conducted on STZ-induced diabetic hamsters. Treatment with BBR at a dosage of 150 mg/kg/d for nine weeks reduced blood glucose, HOMA-IR, body weight, total visceral white adipose tissue weight, adipocyte size, FFAs, TC, LDL-c, TGs, serum leptin, TNF-a, and IL-6 [21]. In addition, oral administration of 100 mg/kg/day BBR for seven weeks decreased FBG, plasma-free fatty acids, C-reactive protein (CRP), triglycerides (TGs), and total cholesterol (TC) and improved glucose tolerance in STZ-induced diabetic rats [22].

A study illustrated that the combination of BBR and stachyose regulated the microbiota flora, increased *Akkermansia muciniphila* abundance and fumaric acid levels, and reduced the metabolite all-transheptaprenyl diphosphate in *db/db* mice. Therefore, fumaric acid and the metabolite all-transheptaprenyl were strongly correlated with *A. muciniphila* [25].

#### 3.2.2. Protective Effects of Berberine against Alloxan-Induced DM

In alloxan-induced diabetic mice with renal impairment, nuclear factor-kappa B (NF-κB) was reduced after treatment with BBR at 300 mg/kg/day. The reduced IB-level worsening was partially recovered. In contrast to the diabetic model group, BBR reduced fibronectin, transforming growth factor (TGF)-β1, and intercellular adhesion molecule-1 (ICAM-1). The inhibitory effects of berberine on the NF-κB signaling pathway may explain why it has an ameliorative impact on extracellular matrix formation [26].

#### 3.2.3. Protective Effects of Berberine against HFD-Induced DM

A study demonstrated that oral administration of 380 mg/day of BBR to rats fed with high-fat food for two weeks reduced body weight. Furthermore, BBR reduced plasma triglycerides and insulin resistance in animals with a high fat content [12]. In another study, 100 mg/kg/day dihydro berberine (a BBR derivative) enhanced the in vivo efficacy of BBR in terms of suppression of increased adiposity, triglyceride accumulation in tissues, and insulin resistance. This finding is likely due to the optimized oral bioavailability of BBR [27].

Another study showed that 5 and 10 mg/kg/day of BBR administration for four weeks significantly reduced body weight, insulin, and the HOMA-IR index without altering food intake in high-fat diet-fed mice [28]. Another study demonstrated that oral administration of 100 mg/kg/day of BBR for four weeks reverted FBGs to normal levels and also decreased the levels of HbA1c, triglycerides and phospholipids, leptin, and insulin in high-fat diet-fed rodents [29].

A study evaluated the effects of rosiglitazone (RSG) co-crystallized with BBR (RB) on diabetic factors in high-sugar, high-fat diet (HSHFD)-induced diabetic KKAy mice. The RB dosages were 0.7 (RB-L), 2.11 (RB-M), and 6.33 mg/kg/day (RB-H). The positive control groups were treated with rosiglitazone 1.04 mg/kg (RSG), BBR 195 mg/kg, or a combination of 1.04 mg/kg RSG and 1.08 mg/kg BBR (MIX). RB significantly reduced FBG, HOMA-IR, white fat index, TG, LDL, and gastric inhibitory polypeptide (GIP) levels in the peripheral circulation and increased insulin sensitivity index (ISI), HDL, and GLP-1 levels. To date, it has been found that the effects due to a concentration of 6.33 mg/kg RB (RB-H) are to some extent superior to those of RSG, BBR, or their mixture. There is probably an explanation for this, i.e., the formation of cocrystals that improve the physical and chemical properties of the drug, as well as its bioavailability. Therefore, RB had more therapeutic benefits than both RSG and BBR in T2DM [30].

In an in vivo experiment, high-fat diet-fed rats were administered 150 and 300 mg/kg/day of BBR for 12 weeks. BBR reduced body weight; urine volume; FBG, blood urea nitrogen (BUN), TC, and hepatic index levels; and pathologic changes and improved ALB levels and upregulated PPM1B in diabetic rats [31].

Li et al. [32] evaluated the effect of BBR alone and a combination of BBR and stachyose in Zucker diabetic fatty rats. The results demonstrated that both BBR alone and in combination had a beneficial effect on diabetes, increased the abundance of beneficial *Akkermansiaceae*, and reduced the abundance of pathogenic *Enterobacteriaceae*. Combined therapy had a more significant impact on reducing the abundance of *Desulfovibrionaceae* and *Proteobacteria* than BBR alone. Furthermore, combination therapy reduced the expression of intestinal early growth response protein 1 (Egr1) and heparin-binding epidermal growth factor (EGF)-like growth factor (HB-EGF). This effect was not observed with BBR alone. Combination therapy (BBR 100 mg/kg/d + stachyose 200 mg/kg/d) also improved glucose metabolism, the balance of α- and β-cells, and mucin-2 expression in T2D *db/db* mice. This effect was more significant in combination therapy than that of BBR alone [32].

### 3.3. Effects of Berberine on Insulin Resistance and Secretion

Interestingly, BBR acted like insulin and improved insulin resistance in *db/db* mice. It stimulated glucose uptake by 3T3-L1 adipocytes at concentrations of 1.25 and 2.5 µM and glucose uptake by L6 myocytes at 2.5–5 µM. BBR also suppressed the phosphatase activity of protein tyrosine phosphatase 1B. In addition, phosphorylation of insulin receptors, insulin receptor substrate1, and Akt were increased by BBR in 3T3-L1 adipocytes [24].

BBR increased the expression of insulin receptor proteins in cultured human liver cells (HepG2) and L6 myocytes through the activation of the insulin receptor gene promotor protein kinase C (PKC)-dependent promoter [20].

In HepG2 cells, BBR up-regulated the expression of alpha7 nicotinic acetylcholine receptor (α7nAChR) protein and suppressed the AChE enzyme. It also showed an anti-inflammatory effect by reducing the pIKKβ Ser181/IKKβ ratio, NF-κB p65 expression, and IL-6 levels. Through this mechanism, BBR could improve insulin resistance [34].

Using a dose-dependent approach, BBR inhibited respiration in L6 myotubes and muscle mitochondria, mirroring the effects of metformin and rosiglitazone on respiratory complex I. Through increased AMPK activity, Respiratory Complex I is a primary target for compounds that improve overall insulin sensitivity. AMPK activation by BBR was not contingent on LKB1 or CAMKKβ activity, indicating a primary regulation at the level of the AMPK phosphatase [27].

In a mouse β-cell line (NIT-1), BBR reversibly inhibited the insulin gene promotor, reducing insulin mRNA and proteins. This effect of BBR was due to its ability to activate AMPK and its downstream molecular phosphorylation of ACC (Acetyl-CoA carboxylase). Consistent with this, treatment with BBR at a dosage of 50 mg/kg/d for ten weeks reduced insulin contents in the islets of HFD-fed mice and improved insulin resistance [35].

BBR improved the insulin resistance of visceral white adipose tissue in T2D hamsters by increasing liver X receptors (LXRs) and PPARs and decreasing sterol regulatory element-binding proteins (SREBPs) [21].

To evaluate the effect of BBR on insulin secretion, BBR was orally administered to BALB/c mice at bolus doses of 93.75, 187.5, and 562.5 mg/kg. Two hours after dosing, serum insulin levels increased (27.5 ± 2.7 or 29 ± 4 or 29 ± 4 vs. 24.3 ± 2.8 pIU/L) and blood glucose decreased (4.52 ± 0.31 or 4.45 ± 0.29 or 4.30 ± 0.19 vs. 4.87 ± 0.21 mM). In another set of experiments, insulin-secreting cells (HIT-T15) and pancreatic islets were incubated with BBR (1–100 μM) for 12 h. Based on the results, BBR at 1–10 μM improved the insulin secretion of mouse HIT-T15 cells and pancreatic islets in a dose-dependent manner [17].

Administration of 100 mg/kg/day of BBR improved many factors associated with insulin resistance in STZ-induced diabetic rats. It was shown that BBR inhibited tyrosine phosphatase-1B (PTP-1B) and dipeptidyl peptidase-4 (DPP-4) dose-dependently, both of which have key roles in glucose metabolism. The IC_50_ values for DPP-4 and PTP-1B were 67 μM and 205 μM, respectively. Inhibition of DPP-4 and PTP-1B is thought to be involved in the insulin-sensitizing effect of BBR [22].

To further understand how BBR works, the researchers looked at its effect on HFD-fed mice and palmitic acid-treated HepG2 cells. BBR (5 and 10 mg/kg/day) was found to reduce hepatic insulin resistance by inhibiting hepatic glycogen synthesis, inhibiting miR-146b, and improving SIRT1 expression by deacetylating FOXO1 through its actions on miR-146b synthesis and inhibition [28]. The importance of SIRT1 in regulating the energy production of cellular systems to meet a cell’s energy requirements has been discussed in detail in the following publications [36,37]. SIRT1 is a nicotinamide adenine dinucleotide (NAD)+ dependent deacetylase that regulates energy production to meet the energy demands of a cell. Noteworthy is the fact that SIRT1 activation is beneficial for T2DM [36,37]. For example, by increasing SIRT1, BBR may be able to reduce oxidative stress in islets of diabetic mice due to increased SIRT1 levels [38]. SIRT1 also plays a crucial role in the regulation of hepatic metabolism. It regulates the activity of FOXO1 by deacetylating FOXO1 to moderate oxidative stress [28]. This study showed that SIRT1 activity was down-regulated in liver tissue from mice fed a high-fat diet (HFD) and in HepG2 cells that had been treated with palmitic acid. Under the circumstances of insulin resistance, palmitic acid-treated HepG2 cells reduced hepatic glycogen synthesis, but BBR (10 μM) administration significantly reformed the impaired hepatic glycogen synthesis by regulating the SIRT1/FOXO1 pathway [28]. In another study, BBR (100 mg/kg/day) was also shown to induce mitochondrial sirt3 activity, improve mitochondrial function, and prevent the deterioration of mitochondrial function caused by impaired oxidative phosphorylation. Thus, the protective effects of BBR may be based on its ability to increase mitochondrial SirT3 activity [29,30,31,32,33,34].

Using in vitro studies, BBR was shown to reduce leukotriene B4 (LTB4)-induced intracellular insulin resistance and inflammation in liver cells. It should also be noted that BBR reduced chemotaxis and inflammatory responses of LTB4-activated macrophages. There was a significant decrease in M1 macrophage gene expression by BBR, which was significantly increased by LTB4 [39]. The possible mechanism of BBR could be related to its effect on the LTB4–BLT1 axis, known as a target for treating metabolic diseases [40]. As a consequence of this interaction, BBR mediated the down-regulation of p-NF-κB expression in macrophages caused by LTB4 [39]. One of the mechanisms of the anti-inflammatory effect of BBR was the MyD88/NF-κB pathway [33]. The MyD88/NF-κB pathway has also been seen as the downstream pathway of the LTB4 pathway [41]. On the other hand, interestingly, LTB4 can influence insulin signaling and inflammation through leukotriene B4 receptor 1 (BLT1), which is often expressed on the surface of liver cells and macrophages. Indeed, the inhibition of BLT1 could suppress the chemotaxis and tracking of macrophages and other immune cells in metabolic tissue, as well as the development of inflammation–insulin resistance syndrome [39].

Another study investigated the effect of rosiglitazone (RSG) co-crystallized with BBR (RB) on insulin resistance in vivo and in vitro. The results showed that RB improved insulin resistance and glucose tolerance via up-regulating PI3K/AKT signaling and inhibiting TXNIP expression in diabetic KKAy mice [30]. These effects may be related to increased BBR dissolution of BBR into RB, which can improve intestinal flora [42].

According to a recent study, co-crystallization of rosiglitazone with BBR (RB) was found to lead to improved glucose uptake, glycogen content, and glucose consumption in insulin-resistant hepatocytes (HepG2). It is also worth mentioning that RB reduces hepatic steatosis and improves glucose and lipid metabolism in the liver. Aside from that, RB prevented the livers and pancreases of mice showing histopathological changes associated with diabetes. This may have been related to improved systemic insulin resistance and glucolipid metabolism [30].

In another in vivo and in vitro study, a dosage of 160 mg/kg/day was administrated to STZ-induced diabetic mice by oral gavage for four weeks, and palmitic acid-induced MIN6 cells were cultured with different concentrations of BBR (2.5, 5, 10, and 20 μM). The results demonstrated that BBR improved β-cell dysfunction and led to improved insulin synthesis. Regarding the mechanism, it has been shown that there is a correlation between miR-204 and β-cell dysfunction, and therefore miR-204 might be the upstream regulatory target of SIRT1. Thus, BBR could improve β-cell dysfunction by reducing miR-204 levels and increasing SIRT1 expression [43].

Another experiment evaluated the effect of BBR on insulin resistance-HepG2 cells (IR-HepG2). Cellular models of IR-HepG2 were constructed by insulin treatment. Then, the IR-HepG2 cells were incubated with different concentrations (5, 10, and 20 μg/mL) of BBR for 24 h. BBR improved glucose consumption, uptake, and inflammation induced in IR-HepG2 cells. The results showed that BBR could suppress insulin resistance via up-regulation of mRNA and protein expression of PPM1B, PPAR-γ, low-density lipoprotein receptor-related protein 1 (LRP1), GLUT-4, insulin receptor substrate 1 (IRS-1), IRS-2, PI3K, AKT, and IKKβ and inhibition of the phosphorylation of pIKKβ Ser181, total IKKβ, NF-κB p65, and JNK in the liver. PPM1B may inhibit insulin resistance in T2D. Therefore, PPM1B may be a downstream target of cyclic AMP (cAMP)/protein kinase A (PKA) signaling, leading to the alleviation of diabetes-related symptoms [31].

In insulin resistance-HepG2 cells (IR-HepG2), the effect of the combination of Astragalus’ polysaccharide (AP) and BBR (BBR) was studied (1:1 mass ratio of AP: BBR). A dose of 10 mg of AP-BBR has been shown to reduce insulin resistance by regulating the gluconeogenesis signaling pathway. It also reduced intracellular H_2_O_2_ without any significant effect on the apoptosis of IR-HepG2 cells. Furthermore, the intracellular calcium current was changed, and AP-BBR significantly reduced this change. AP-BBR also reduced the increased protein expression of p-FoxO1 and PEPCK and increased the decreased expression of GLUT2 protein [44].

The effect of BBR was also studied in adipocytes with resistance to insulin-3T3-L1. Insulin-resistance models of 3T3-L1 adipocytes were constructed with 1 μM dexamethasone and 1 μM insulin. BBR treatment increased the utilization frequency of glucose and adiponectin secretion and reduced fat deposits. It improved insulin resistance by increasing the expression of hypoxia-inducible factor-3α (HIF3A) and reducing HIF3A methylation, in which IRS-1 and GLUT4 expressions were positively correlated with the concentrations of BBR [45].

In another study, BBR (200 mg/kg/day) improved insulin resistance by reducing the abundance of branched-chain amino acid (BCAA)-producing bacteria (the families *Streptococcaceae, Clostridiaceae*, and *Prevotellaceae* and the genera *Streptococcus* and *Prevotella*) in HFD-fed mice. Consistent with this, BBR reduced BCAAs in AML12 hepatocytes and 3T3-L1 adipocytes [46].

### 3.4. Protective Effects of Berberine against Diabetes Complications

DM is an endocrine disorder that can lead to many chronic complications, including osteoporosis, retinopathy, nephropathy, neuropathy, cardiovascular diseases, and hepatic disorders. Interestingly, protective properties of BBR against DM complications have been reported (Table 3 and Table 4).

#### 3.4.1. Diabetes-Induced Osteoporosis

One of the complications of diabetes mellitus is osteoporosis. Diabetes suppresses osteogenesis and compromises the osseointegration process, causing dental-implant failure. Dental implants are used worldwide to treat dentition defects, but there is a major problem for diabetic patients [87].

To study the effect of BBR on implant recovery, 120 mg/kg/day of BBR was administrated (gated for four weeks) to STZ-induced diabetic rats and was also added to high-density bone mesenchymal stem cells (BMSCs) with medium glucose contents. The results showed that BBR improved glucose and bone metabolism in diabetic rats through the ROS-mediated IRS-1 signaling pathway. Furthermore, in BMSCs, BBR increased osteogenesis and up-regulated the ROS-mediated IRS-1 signaling pathway. Therefore, BBR administration could be a good candidate for diabetic patients requiring an implant [75].

#### 3.4.2. Diabetes-Induced Gut Microbiota Alteration

The microbiota is considered a functional organ, so its composition affects the host’s glycemic control system [50]. For example, one of the microbiota components related to diabetic chronic inflammation is lipopolysaccharide (LPS), a component of Gram-negative bacteria cell walls [3].

Treatment with 100 mg/kg/day of BBR for three weeks inhibited the progression from prediabetes to diabetes in 70% of diabetic fatty rats by restoring an average diversity of gut microbiota and increasing fasting plasma GLP-2 and glutamine-induced intestinal GLP-2 secretion. In this experiment, BBR reduced food intake, FBG, insulin resistance, and LPS levels but increased the number of goblet cells and villi length. Furthermore, it increased the expression of mucins and major tight junction proteins, namely, occludin and zona occludens-1 (ZO-1), and down-regulated the expressions of TLR4, NF-κB, and TNF-α [73].

To study the effect of BBR on alterations in the gut microbiota, diabetic rats were divided into two groups: one group received BBR (200 mg/kg/day) + antibiotics (100 μg/mL metronidazole, 25 μg/mL vancomycin, and 50 μg/mL neomycin in sterile water daily); the other group received BBR only. The results demonstrated that BBR reduced FBG and improved glucose tolerance and serum lipid parameters. Therapeutic effects of BBR were observed in both groups. In contrast, these effects were weaker in the BBR + antibiotics (BA) group than in the BBR group. Therefore, BBR may influence the gut microbiota, which may be its mechanism for alleviating diabetes. The number and variety of intestinal flora increased significantly after BBR administration. BBR increased the abundance of Bacteroidetes and reduced that of Proteobacteria and Verrucomicrobia. In both the BBR and BA groups, *Lactobacillaceae* increased considerably, negatively influencing the risk of T2DM. It was shown that 55 intestinal metabolites differed between the BBR and model groups. In the BBR group, some aromatic amino acids in the serum and colon were significantly decreased [74].

A combination of BBR with oryzanol and vitamin B6 showed an antidiabetic effect. This combination increased *Bacteroidaceae* and *Clostridiaceae* in in diabetic *db/db* mice and reduced FBG and HbA1c more than BBR therapy alone in diabetic mice. The increase in the mentioned microbiome leads to more secondary bile acid, deoxycholic acid (DCA), from primary bile acid and cholic acid (CA). Furthermore, DCA increased TGR5 and GLP, which led to improved metabolism of carbohydrates, lipids, amino acids, and nucleotides [76].

#### 3.4.3. Diabetic-Induced Hepatic Damage

An experiment was conducted to determine whether BBR could ameliorate T2D-associated hepatic gluconeogenic and lipid metabolism disorders in STZ-induced diabetic mice and palmitic acid-treated HepG2 cells. BBR treatment reduced levels of HNF-4α and expression of miR122; the key gluconeogenesis enzymes, namely, phosphoenolpyruvate carboxykinase (PEPCK) and glucose-6-phosphatase (G6Pase); and key enzymes and proteins in lipid metabolism, such as SREBP-1, fatty acid synthase-1 (FAS-1), and acetyl-coenzyme A carboxylase (ACCα), but increased carnitine palmitoyltransferase-1 (CPT1) in both diabetic mice and palmitic acid-treated HepG2 cells. MicroRNA 122 is an essential hepatocyte nuclear factor 4α (HNF4α) regulator in the regulation of hepatic gluconeogenesis and lipid metabolism in HepG2 cells. So, the protective effect of BBR on hepatic gluconeogenesis and lipid metabolism disorders was mediated by HNF-4α and maintained downstream of miR122 [66].

#### 3.4.4. Diabetic Retinopathy

BBR showed a protective effect against diabetic retinopathy by activating the AMPK/mTOR signaling pathway [77,88] and inhibiting Akt/mTOR-mediated hypoxia-inducible factor (HIF)-1α/vascular endothelial growth factor (VEGF) activation [8]. In diabetic patients, normal LDL changes to highly oxidized and glycated (HOG)-LDL. This type of LDL damages retinal cells, leading to diabetic retinopathy [89]. In an in vitro study, BBR was added to HOG-LDL-induced human retinal Muller cells. The results showed that BBR activates AMPK and could increase cell viability (reducing HOG-LDL-induced cytotoxicity, autophagy, and apoptosis). BBR decreases oxidative stress, the expression of angiogenic factors, inflammation, and glial fibrillary acidic (GFA) protein expression. In conditions of retinal damage, GFA is over-expressed; therefore, it is considered a significant factor in the development of retinopathy [88].

In high-glucose-induced rat retinal Müller cells, BBR reduced apoptosis and the expression of Bax and caspase-3 and increased the expression of Bcl-2. Furthermore, BBR increased autophagy, which was inhibited by high-glucose conditions. Consistent with this, the expression of autophagy markers (Beclin-1 and LC3II) was also increased. The results also demonstrated that BBR enhances the AMPK/mTOR signaling pathway [77].

To study the anti-DR effect of BBR in animal models, BBR was administrated orally (50 and 100 mg/kg) and via ocular delivery (0.2 and 0.4 μg/kg) to types I and II diabetic mice which were treated with insulin. BBR was found to significantly inhibit the expression of VEGF and HIF-1α in retinal endothelial cells. In addition, it also inhibited the Akt/mTOR signaling pathway. Furthermore, BBR also inhibited insulin-induced retinal neovascularization. Finally, it was demonstrated that BBR inhibited the progression of DR in both types I and II diabetic mice subjected to insulin therapy [8].

#### 3.4.5. Diabetic Vascular Complications

Endothelial dysfunction plays a major role in the onset of vascular problems due to diabetes. Advanced glycation end products (AGEs) have been linked to endothelial dysfunction through various mechanisms and excessive glucose [90,91].

To simulate clinical circumstances, researchers created an in vitro model of diabetic micro-endothelial (microEC) damage caused by the combination of high glucose and AGEs. The results showed that BBR treatments significantly increased the synthesis of thrombomodulin, NOS, and NO. Additionally, BBR was found to have potent inhibitory effects on AGE production [78].

In cultured endothelial cells and blood vessels isolated from rat aorta, BBR ameliorated high-glucose-induced endothelial dysfunction by increasing eNOS and NO and decreasing glucose-induced ROS, cell apoptosis, NF-kB activation, and the expression of adhesion molecules, which led to inhibition of the attachment of monocytes to endothelial cells. Furthermore, BBR increased endothelium-dependent vasodilatation through activation of AMPK [79].

Moreover, BBR (200 mg/kg/day, gavage for four weeks) improved insulin sensitivity of mesenteric arteries and showed protective effects against endothelial dysfunction in STZ-induced diabetic rats through up-regulation of receptor-mediated insulin signaling. Furthermore, it improved vasodilatation, and its vasodilator effects were mediated through a PI3K/Akt-dependent mechanism. In the ex vivo part of this experiment, BBR (2.5–10 μM) in combination with a low insulin concentration significantly improved impaired vasodilatation in isolated mesenteric artery loops of diabetic rats, which suggests synergistic effects between insulin and BBR. The combined therapy of insulin and BBR has also been shown to increase the phosphorylation of InsR, AMPK, Akt, and eNOS. Thus, BBR enhances endothelium-mediated vasodilatation through a mechanism involving both Akt and AMPK activation [67].

An in vitro study demonstrated that BBR improved palmitate-induced endothelial dysfunction in human umbilical vein endothelial cells (HUVECs). This was due to BBR increasing the expression of eNOS and reducing nicotinamide adenine dinucleotide phosphate (NADPH) oxidase 4 (NOX4) protein expression. In addition, BBR increased the protein expression of AMPK and p-AMPK, which might be related to its effect on eNOS and NOX4. It also increased NO and decreased ROS production. No effect of BBR on Akt was observed in this study [80].

Hyperglycemia increases Ca^2+^, causes smooth muscle contraction, and leads to diabetic vascular dysfunction [63]. Therefore, research was conducted to evaluate the effects of 50, 100, and 200 mg/kg/day of BBR on cerebrovascular contractile function independent of a functional endothelium in STZ-induced diabetic rats. The results showed that chronic treatment with 100 mg/kg/day BBR reduced glucose levels and inhibited increase in cerebral artery contractile function by blocking L-type Ca^2+^ channels and suppressing Ca^2+^ release in cerebral vascular smooth cells isolated from T2D rats. Similarly, 10 μM BBR directly inhibited hyperglycemia-induced L-type Ca^2+^ channel currents and suppressed hyperglycemia-induced Ca^2+^ release in cerebral vascular smooth cells isolated from normal control rats [63].

#### 3.4.6. Diabetic-Induced Neuropathy

BBR improved cold and mechanical allodynia at doses of 10 and 20 mg/kg (single and repeated intraperitoneal injection, twice daily for 14 days) in a rat diabetic neuropathy model. A dose of 5 mg/kg of BBR was insufficient to significantly reduce allodynia. Diabetes increased hepatic MDA, SOD, catalase, and GPx activities, while BBR administration reduced all of these factors in a dose-dependent manner. The antioxidative effects of 10 mg/kg BBR were quite similar to those of 10 mg/kg amitriptyline. A dosage of BBR 20 mg/kg showed antiallodynic results identical to those obtained with a dosage of 10 mg/kg amitriptyline. Therefore, the antiallodynic effect of BBR is assumed to be related to its antioxidative effects [14]. In another study, BBR improved mechanical allodynia and thermal hyperalgesia by developing a mechanical threshold and thermal latency in STZ-induced diabetic mice. It inhibited the activations of microglia and astrocytes in the spinal cord and also inhibited the expression of pro-inflammatory cytokines (TNF-α, IL-6, and IL-1β) and inflammatory proteins (iNOS and COX-2). Therefore, the mechanism of the antinociceptive effect of BBR on diabetic neuropathic pain is related to its ability to suppress neuroglia activation and inflammation [59].

In another study, 5, 20, and 40 mg/kg/day of BBR were administered to STZ-induced diabetic rats for ten weeks. A dosage of 5 mg/kg/day was insufficient to show a protective effect, but doses of 20 and 40 mg/kg/day had a beneficial impact on diabetes and neuropathic pain. BBR increased the threshold of mechanical and thermal nociception. It reduced ROS and MDA and increased catalase activity, while insulin therapy did not inhibit excessive oxidative stress. Furthermore, BBR suppressed neuroinflammation by reducing TNF-α and IL-6. BBR also up-regulated μ-opioid receptor (MOR) expression [63]. It should be mentioned that neuroinflammation markers were suppressed in response to MOR up-regulation. In this way, MOR plays a crucial role in diseases related to chronic inflammation, such as diabetes [92].

In high-glucose SH-SY5Y human neuroblastoma cells, BBR showed a protective effect against diabetic neuropathy through activation of nuclear erythroid 2-related factor 2 (Nrf2), an essential antioxidative transcription factor, which leads to up-regulation of heme oxygenase-1 (HO-1) and nerve growth factor (NGF). BBR inhibited high-glucose-induced neuronal apoptosis by reducing cytochrome C release and increasing the expression of the anti-apoptotic protein Bcl-2 [81].

#### 3.4.7. Diabetic-Induced Nephropathy

Kumaş et al. [72] investigated the effect of 50, 100, and 150 mg/kg/day of BBR on STZ-induced diabetic rats with renal ischemia/reperfusion injury. The results showed that the dosage of 50 mg/kg/day was insufficient to produce sufficient protective effects. Dosages of 100 and 150 mg/kg/day significantly improved renal function and reduced the elevation of BUN and creatinine levels. BBR rearranged the intercellular ion concentration by increasing the decreased activity of Ca^2+^-ATPase and Na^+^/K^+^-ATPase enzymes in diabetic rats. All doses of BBR (50, 100, and 150 mg/kg/day) reduced enzyme lactate dehydrogenase (LDH) levels, a marker of tubular necrosis. In general, BBR improves diabetic-induced nephropathy through its antioxidant, anti-inflammatory, and antiapoptotic properties [72].

BBR doses of 100 mg/kg and 200 mg/kg were administered to STZ-induced diabetic nephropathic golden hamsters for eight weeks. Consequently, blood glucose, blood lipids, and renal function were improved and the expression of inflammatory factors (IL-1β and IL-6), NOD-like receptor pyrin domain-containing protein 3 (NLRP3), caspase-1, and Gasdermin D (GSDMD); the number of TUNEL-positive cells; and MDA levels were decreased; however, Nrf2 expression was increased [68]. Briefly, BBR inhibited pyroptosis and diabetic nephropathic damage by regulating Nrf2 and NLRP3-Caspase-1-GSDMD signaling [68].

Another study investigated the effect of BBR on STZ-induced diabetic nephropathic rats and a high-glucose-induced human renal proximal tubular epithelial cell line (HK-2). The results showed that treatment (150 mg/kg/d for 12 weeks) improved kidney function. In both animal and cell models, epithelial-to-mesenchymal transition (EMT) was suppressed, and the NOD-like receptor pyrin domain-containing protein 3 (NLRP3) inflammasome was down-regulated by BBR [69].

Another in vitro experiment demonstrated that BBR (25 µM) protected palmitate-induced lipid accumulation and apoptosis in HK-2 cells. The protein expressions of CPT1A, PPAR-α, and peroxisome proliferator-activated receptor γ coactivator-1α (PGC-1α) were up-regulated in HK-2 cells treated with or without palmitate, and intracellular lipid accumulation and apoptosis were reversed through the promotion of fatty acid oxidation by BBR treatment [82].

Another experiment evaluated the effect of BBR (200 and 300 mg/kg/day) on diabetic *db/db* mice and palmitic acid-induced cultured podocytes. Disordered metabolism, podocyte damage, and glomerulosclerosis were improved by BBR treatment in mice. In addition, BBR inhibited lipid accumulation, excessive generation of mitochondrial ROS, mitochondrial dysfunction, and deficient fatty acid oxidation in mice and podocytes. These reno-protective effects of BBR have been associated with its ability to restore the PGC-1α signaling pathway, which promotes mitochondrial energy homeostasis and fatty acid oxidation in podocytes [83].

In another in vivo study, excessive oxidative stress, glomerulosclerosis, and abnormal kidney function were observed in STZ-induced diabetic mice. It was shown that BBR treatment (200 mg/kg/day for eight weeks) improved renal function through activation of AMPK in diabetic mice. In a series of in vitro experiments, BBR increased the activity and phosphorylation of thr172, leading to dose-dependent activation of AMPK in cultured human glomerulus mesangial cells (HGMCs). It was also shown that LKB1 (an AMPK upstream kinase) appears to be necessary for AMPK activation. Under high-glucose (30 mM) conditions, BBR demonstrated an antioxidant effect and significantly suppressed excessive oxidative stress [70].

BBR could reduce diabetic renal fibrosis by suppressing RhoA/ROCK signaling and reducing NF-κB activity, leading to a decrease in the inflammatory factor ICAM-1, the cytokine TGF-β1, and fibronectin overexpressed in the kidneys of diabetic rats and high-glucose-induced glomerular mesangial cells (GMCs). In addition, BBR also reduced high-glucose-induced excessive reactive oxygens in GMCs [71].

In another study, the primary mechanism underlying the fibrosis-reducing effect of BBR was the activation of Takeda G protein-coupled receptor 5 (TGR5, alternatively named the G protein-coupled bile acid receptor 1 (GPBAR-1)) and inhibition of sphingosine 1-phosphate receptor 2 (S1P2)/MAPK signaling, leading to a decrease in fibronectin, the inflammatory factor ICAM-1, and the cytokine TGF-β1 and inhibition of the phosphorylation of the nuclear factor AP-1 heterodimer c-Jun/c-Fos in high-glucose-induced GMCs [84]. Indeed, TGR5 is a bile acid receptor that can prevent renal disorders by reducing renal oxidative stress and lipid accumulation [93]. Therefore, BBR is able to attenuate high-glucose-induced fibrosis via a different mechanism, such as inhibiting TGR5.

#### 3.4.8. Diabetic-Induced Cardiovascular Disease

To investigate the cardioprotective effect of BBR against ischemia/reperfusion (I/R) in diabetic rats, diabetes was induced in rats for 12 weeks. Then, saline or BBR (100, 200, and 400 mg/kg/d) was administered intragastrically to diabetic rats, starting from weeks 9 to 12. At the end of the period of 12 weeks, myocardial ischemia and reperfusion were induced in all rats. The results showed that BBR significantly helped the recovery of systolic/diastolic cardiac function and reduced myocardial apoptosis through the activation of AMPK and PI3KAkt–eNOS signaling in the mentioned animals [13]. As in the above study, 100 mg/kg BBR was administered to STZ-induced diabetic rats for seven days before the I/R induction. This BBR pretreatment reduced I/R injury and decreased arrhythmia in diabetic rats. BBR reduced the serum levels of TGs, TC, and MDA, while it did not change the serum levels of FBG and SOD. This study demonstrated that the cardioprotective mechanism of BBR leads to the inhibition of glycogen synthase kinase 3β (GSK3β) and increases AKT phosphorylation and AMP/ATP and ADP/ATP ratios, which in turn lead to an increase in AMPK in non-ischemic areas [94].

Administration of BBR at a dose of 100 mg/kg for seven days could alleviate ischemic arrhythmias in STZ-induced diabetic rats. It reduced the time of prolonged QTc intervals from 214 ± 6 ms to 189 ± 5 ms and restored K+ currents and L-type Ca^2+^ currents to their normal states in diabetic rats [47]. Furthermore, dosing of 180 mg/kg/day BBR for 14 days also had antiarrhythmic effects by recovering K^+^ currents and current density and increasing the decreased Kir2 (the main K^+^ channel subunit that mediates K^+^ currents) in STZ-induced diabetic rats with myocardial infarction [10].

In another study, diabetes mellitus was induced in pregnant mice and then the effect of BBR on the function of the cardiac mitochondria and mitochondrial phospholipid cardiolipin of the newborn mice was investigated. To induce gestational diabetes, female mice were fed a high-fat diet for six weeks before reproduction. Another group of pregnant mice was fed a low-fat diet. Lean male offspring and male offspring exposed to gestational diabetes were randomly assigned to a low-fat diet, a high-fat diet, or a high-fat diet containing BBR (160 mg/kg/day) at weaning for 12 weeks. The results showed that the expression of the cardiolipin remodeling enzyme (tafazzin) increased in male offspring exposed to gestational diabetes and fed a diet containing BBR, followed by an increase in the amount of tetra-linoleic-cardiolipin and total cardiolipin in the heart. These descendants also showed increased expression of cardiac enzymes involved in fatty acid uptake, oxidation, and electron transport chain subunits. Moreover, BBR reduced elevated plasma levels of non-esterified fatty acids (NEFAs) in offspring from lean mothers fed a high-fat diet. It also reduced plasma TG levels in lean offspring fed a high-fat diet and triacylglycerol accumulation in the heart, skeletal muscle, and liver of GDM-exposed offspring [48]. Furthermore, BBR significantly reduced elevated plasma ketone levels in offspring fed a high-fat diet and exposed to gestational diabetes mellitus [48].

To study the effect of BBR on cardiac remodeling, 200 mg/kg/day of BBR was orally administrated to STZ induced-diabetic rats for four weeks. Cardiac matrix collagen deposition and dysfunction have been observed in diabetic rats. Furthermore, IGF-1R expression was up-regulated in cardiac fibroblasts isolated from the hearts of diabetic rats or cultured under hyperglycemia conditions (30 mM). IGF-1R induced cell differentiation and collagen production through up-regulation of matrix metalloproteinase-2 (MMP-2)/MMP-9 expression, smooth muscle actin, and collagen type I in cardiac fibroblasts. BBR treatment significantly reversed all these effects and inhibited all the mentioned factors, thus exerting an antifibrotic influence on both diabetic myocardium and high-glucose-cultured cardiac fibroblasts. Overall, BBR may have the potency to treat diabetic cardiomyopathy associated with cardiac fibrosis [51].

In another study, 100 mg/kg/d BBR was administrated orally to STZ-induced diabetic cardiomyopathic rats for an additional 16 weeks. BBR administration improved cardiac function and attenuated cardiac hypertrophy, fibrosis, and parameters that were associated with cardiac fibrosis (reduced collagen deposition and TGF-β expression). Consistent with this, H9c2 cells incubated with palmitate and treated with BBR showed reduced hypertrophy, increased α-MHC expression, and decreased β-MHC expression. BBR also increased AMPK and AKT activation and decreased GSK3β activation in both palmitate-incubated H9c2 cells and STZ-induced diabetic cardiomyopathic rats [95]. In insulin-resistant rat H9c2 cardiomyocytes, BBR could reduce insulin resistance and increase glucose consumption and absorption by stimulating AMPK activity [85].

One study evaluated the effect of BBR on hypertension and vascular function in STZ-induced diabetic rats. Doses of 50, 100, and 200 mg/kg/day of BBR were administrated for eight weeks. The results showed that chronic intragastric administration of 100 mg/kg/day BBR, in addition to lowering blood glucose and increasing body weight, also lowered systolic and diastolic blood pressure and improved vasodilation in diabetic rats. Administration of 200 mg/kg/day of BBR reduced blood pressure and increased vascular relaxation in both diabetic and control-group rats. A 50 mg/kg/day dose of BBR showed no significant effect. Furthermore, in cerebral vascular smooth muscle cells (VSMCs) isolated from diabetic rats and in VSMCs induced by high glucose levels, calcium-activated K+ channel (BKCa) β1-subunit expression was increased by BBR treatment. Therefore, it may be possible that BKCa channel activation is the underlying mechanism responsible for the vascular protective effect of BBR in diabetes [52]. In another study, BBR increased glucose uptake and consumption in palmitate-induced insulin-resistant H9c2 cardiomyocytes by activating protein kinase B (AKT) and increasing the expression of the glucose transporter GLUT-4. BBR had beneficial effects on these cells by reducing accumulated DAG and increasing TAG accumulation [86].

Research conducted on high-fat diet-induced diabetic hamsters and human cell lines treated with insulin showed that the combination of BBR and metformin contributed to the suppression of sebocyte apoptosis, which reduced susceptibility to cardiovascular complications of diabetes. This antiapoptotic effect of the combination of BBR and metformin was due to its down-regulating effect on the Bik protein [53].

Gut microbiota alteration is closely linked to atherosclerosis [96,97]; therefore, BBR showed an antiatherosclerosis effect by modifying the intestinal microbiota and inhibiting systematic inflammation [73,98]. In addition, BBR reduced the abundance of Akkermansia spp. and Bacteroides and suppressed arterial and intestinal expression of TNF-a and IL-1b in Apoe−/− HFD-fed mice [49].

In cohoused BBR-treated Apoe−/− HFD-mice with non-BBR-treated Apoe−/− HFD-fed mice, the abundances of Firmicutes and Verrucomicrobia were changed because the mice exchanged their gut microbiota [50]. BBR increased the thickness of the colonic mucus layer, which correlated with the restoration of gut barrier integrity in Apoe−/− HFD-fed mice. Other beneficial effects of BBR were also observed: reduced TC, attenuated HFD-induced metabolic endotoxemia (i.e., LPS), and increased expression of ZO-1 and Occludin in the ileum and colon, respectively. BBR protects against atherosclerosis by lowering VCAM-1 and MMP-2 [49]. Matrix metalloproteinase-2 and vascular cell adhesion molecule (VCAM)-1 induce macrophage adhesion to the vascular endothelium, leading to plaque instability [99,100].

BBR could down-regulate the expression of FMO3, which is responsible for trimethylamine N-oxide (TMAO) production from gut microbial metabolite trimethylamine (TMA). BBR also reduces serum TMAO, which promotes atherosclerosis; therefore, BBR prevents the development of atherosclerosis by inhibiting the TMA-FMO3-TMAO pathway [50].

#### 3.4.9. Diabetes-Induced Central Nervous System (CNS) Disorders

Chronic inflammation and mediators of insulin resistance cause the production of β-amyloid (Aβ)42, a marker of Alzheimer’s disease, in the diabetic brain [9]. Furthermore, memory impairment in diabetes mellitus was mainly associated with glucose uptake/metabolism in the medial prefrontal cortex (mPFC). Intragastric administration of BBR (187.5 mg/Kg/d) ameliorated diabetes-associated memory impairment by modulating the abnormal inflammatory response and improving insulin resistance in the mPFCs of STZ-induced diabetic rats. BBR also reduced the activation of the PI3K/Akt/mTOR and MAPK signaling pathways, as well as two isoforms, PKCη and PKCε, and the translocation of NF-κB in neurons. In addition, GLUT3 was significantly increased in the BBR-treated animals. Furthermore, BBR increased glucose uptake, while decreasing the expressions of amyloid precursor protein and β-site amyloid-precursor-protein-cleaving enzyme 1 (BACE-1 and β-secretase 1) and the production of oligomeric Aβ42. Therefore, BBR has the potency to accelerate information consolidation and improve cognitive impairment in diabetes [9].

Another study investigated the effect of BBR on STZ-induced diabetic rats that had memory impairments. The results showed that oral administration of BBR at 25–100 mg/kg twice daily for 30 days reduced hyperglycemia, oxidative stress, and AChE activity and improved cognitive performance in diabetic rats. In another series of experiments, learning and memory were also improved by administering 100 mg/kg BBR for 30 days [54]. In another study, daily administration of 50 and 100 mg/kg BBR (orally) restored impaired neurochemicals and showed a neuroprotective effect in STZ-induced diabetic rats through reductions in AChE, butyrylcholinesterase (BChE), and MAO and MDA activities and increased SOD, GPx activities, and GSH levels [55]. To study the effect of BBR on diabetic encephalopathy, 50 mg/kg/day of BBR was administrated orally to *db/db* mice. The results showed that BBR improved learning and memory. It also improved lipid metabolism; reduced body weight and FBG, TG, TC, and LDL levels; and increased HDL levels in *db/db* mice. Moreover, BBR increased synapse- and nerve-related protein expression, such as PSD95, SYN, and NGF. Furthermore, it reduced the expression of inflammatory factors such as TNF- κB and NF-κB in the hippocampi of *db/db* mice. In addition, BBR increased the expression of SIRT1 and down-regulated ER stress-related proteins (PERK, IRE-1a, eIF-2a, PDI, and CHOP) in the hippocampi of *db/db* mice. Thus, the SIRT1/ER stress pathway might be the mechanism of the neuroprotective effect of BBR [57].

In STZ-induced Alzheimer’s diabetic rats, BBR alleviated memory impairment by suppressing the ER stress pathway. It decreased major ER stress-related proteins in the hippocampus, eliminated Aβ deposition, restored the disorganized arrangement and damage of nerve cells, and reduced the apoptosis rate of nerve cells, leading to improvement in diabetic Alzheimer’s disease [62]. A dosage of 187.75 mg/kg/day of BBR improved spatial learning memory by inhibiting Aβ formation and reducing CSF/glycemia, inflammatory response, and AChE activity. BBR has been found to increase the expression of α7-nAChRs, inhibiting CNS or peripheral inflammation [65]. Doses of 20 and 40 mg/kg of BBR showed neuroprotective effects against neonatal-STZ-induced diabetic peripheral neuropathy through a reduction in pro-inflammatory cytokines and oxide-nitrosative stress and an increase in the expression levels of BDNF, IGF-1, PPAR-γ, and AMPK. Through these mechanisms, BBR ameliorated impaired allodynia, hyperalgesia, and impaired nerve conduction velocity in neonatal diabetic rats with neuropathy [61,101].

BBR also decreased STZ-induced atrophy in myelinated axons and attenuated mitochondrial alterations [61]. One study demonstrated that BBR at a dosage of 200 mg/kg/day had protective effects against cerebral ischemia/reperfusion injury in diabetic rats by increasing the expression of PI3K and p-Akt. BBR reduced cerebral infarct volume and cell apoptosis of the cerebral infarct area. It decreased NO and MDA and increased SOD. It also up-regulated Bcl-2 and down-regulated the expression of Caspase-3 and Bax. Therefore, BBR has the potential to be used for the prevention or treatment of cerebral ischemic brain disease in diabetes [65]. Another study demonstrated that the combination of BBR (10 mg/kg) with gypenosides (1 mg/kg) and bifendate (a synthetic intermediate of Schisandrin C; 0.3 mg/kg) has an antidiabetic effect in *db/db* and STZ-induced diabetic mice. However, unlike its antidiabetic effects, at these low doses, it showed no positive impact on memory impairment. It should be mentioned that, separately, low-dose BBR, gypenosides, and bifendate were not effective in diabetes; therefore, they might have had a synergistic effect [58].

### 3.5. Clinical Investigations

A clinical trial was conducted with 97 T2DM patients. Fifty patients received BBR (1 g/d orally), twenty-six patients received metformin (1.5 g/d orally), and the others (21 patients) received rosiglitazone (4 mg/d orally) for two months. BBR reduced FBG and HbA1c, as did metformin and rosiglitazone. It reduced TGs more than metformin and rosiglitazone. BBR also decreased the FINS. No adverse effects were observed in the BBR group. Furthermore, blood samples from the BBR group showed a high number of lymphocytes expressing insulin receptors [5].

In another series of experiments, 35 patients with chronic hepatitis and T2DM or impaired fasting glycemia were enrolled. Eighteen patients were infected with HCV, and seventeen had HBV disease. Again, all 35 patients were administered 1g/d of BBR for two months. The results showed that BBR reduced all patients’ FBG, TG, ALT, and AST levels. Furthermore, no adverse effects were observed in the BBR group [5].

In one study, 45 pregnant women with gestational diabetes mellitus (GDM) and 43 healthy controls enrolled in a clinical trial for one year. Subcutaneous adipose tissue was collected from the abdominal region during cesarean delivery. In comparison, the mRNA and methylation levels of hypoxia-inducible factor-3α (HIF3A) in the GDM group were lower than in the control group, and the level of methylated HIF3A was higher in the GDM group [45]. Therefore, there is a high correlation between HIF3A gene methylation and insulin resistance in GDM [64].

In a randomized, double-blinded, placebo-controlled study, 365 participants with T2D were enrolled and randomly divided into four groups: the first group received a BBR dose of 0.6 g (six pills twice a day) plus 4 g of probiotics (two strips of powder once a day) (Prob + BBR), the second group received probiotics plus placebo (Prob), the third group received BBR plus placebo (BBR), and the fourth received placebo plus placebo (Plac) for 12 weeks. The results showed that postprandial TC and LDL levels were reduced more significantly in the Prob+BBR group than in BBR or Prob alone. Furthermore, several types of postprandial lipidomic metabolites were reduced: medium-chain fatty acids (FFAs), acyl-carnitines, and multiple glycerophospholipids: lysoglycerophosphatidylcholine (LPC), lysoglycerophatidylethanolamine (LPE), glycerophosphatidylcholine (PC), and glycerophatidylethanolamine (PE) with alkyl and alkenyl substituents. Fecal samples from the participants showed changes in fecal *Bifidobacterium breve* abundance that could be related to the therapeutic effects of BBR and Prob+BBR. In vitro analysis showed that BBR activated four *fadD* genes encoding long-chain Acyl-CoA synthetase in the *B. breve* strain. Consistent with this, BBR reduced the concentration of FFAs in the medium of *B. breve*, which may be related to its effect on *fadD* genes. Therefore, BBR reduced intraluminal lipids for absorption and synergized with Prob [102].

Another clinical trial was conducted to investigate the effects of long-term adjuvant therapy of BBR on renal damage in 69 hypertensive patients with T2DM. Doses of 0.1 g of BBR were administered three times daily to patients for two years. Every five months, each patient spent two weeks drug-free and on BBR. The patient received standard hypotensive and hypoglycemic treatment before and during the experiment, so they had blood pressure and fasting blood sugar levels monitored. The patients were randomly divided into control (33 patients) and BBR (36 patients) groups. The results showed that biochemical markers of renal damage, including urinary albumin-to-creatine ratio (UACR), urinary osteopontin, and kidney injury molecule-1 (KIM-1), were significantly reduced in the BBR group. In addition, BBR improved renal hemodynamics and reduced inflammation and oxidative stress [103].

A prospective, randomized, double-blind, placebo-controlled trial of two doses of BBR ursodeoxycholate (500 and 1000 mg) was conducted in 100 patients with fatty liver disease and T2D. (Berberine ursodeoxycholate is an ionic salt of BBR and ursodeoxycholic acid.) This experiment lasted 18 weeks. The results showed that the fat content in the liver decreased more in the BBR group (patients received 1000 mg BBR twice daily) than in the placebo group. Furthermore, this BBR group showed significant improvement in glycemic control and liver-associated enzymes significantly decreased, indicating notable weight loss. Concerning the safety of BBR, the most frequently reported adverse effects were diarrhea and abdominal discomfort [104].

In another clinical trial, 300 mg of BBR was administered to thirty T2D patients thrice a day (30 min after each principal meal) for eight weeks. After BBR treatment, biochemical parameters, such as BMI, FBG, FINS, HbAlc, LDL, HDL, TC, and TGs, decreased. In 16 patients, the FBG level was restored to a normal level by BBR. In addition, BBR reduced LPS, CRP, and TNF-α levels in diabetic patients. BBR was found to impact fecal gut microbiota; increase total Bifidobacterium, *B. longum, B. breve*, and *B. adolescentis*; and decrease *B. infantis*. Therefore, it could be concluded that BBR improved T2D by altering the abundance of *Bifidobacterium* species, leading to a reduction in T2D-related inflammation [105].

### 3.6. Toxicity of and Cautionary Notes on Berberine

BBR toxicity varies depending on the amount of BBR contained in a compound, the route of administration, and the type of organism. When orally administered to mice, *Berberis vulgaris* root powder had an LD_50_ value of 2600 mg/kg, *B. vulgaris* root extract had an LD_50_ value of 520 mg/kg, and pure BBR had an LD_50_ value of 329 mg/kg. When administered intraperitoneally to mice, pure BBR had an LD_50_ value of 23 mg/kg [106].

In rats, the LD_50_ value of *B. vulgaris* after oral treatment of the root extract fraction was 1280 mg/kg, and the LD_50_ value of BBR sulfate after IP treatment of the BBR sulfate extracted from *Berberis aristate* was 205 mg/kg. Moreover, 40% of rats experienced diarrhea after receiving 50 mg/kg of BBR sulfate, resulting in an immediate negative impact on the digestive system [106].

Oral administration of 100 mg/kg BBR to cats caused vomiting for 6–8 h, while 100 mg/kg BBR administration for 8–10 days caused the death of all animals. Oral administration of berberine sulfate in cats at doses of 50 or 100 mg/kg for 10 days caused inflammatory bleeding problems in both the small and large intestines [107]. Dogs showed some moderate signs of toxicity at low doses of BBR and related compounds. These signs included salivation, nausea, diarrhea, emesis, muscle tremor, and occasional paralysis [107].

In another study, BBR demonstrated immunotoxic effects. A dose of 10 mg/kg of BBR decreased the numbers of leukocytes, neutrophils, and lymphocytes and reduced spleen weight. It also inhibited the production/development of B- and T-cells and splenic CD19^+^ B-cells and CD4^+^ and CD8^+^ T-cells. Ultimately, 5 mg/kg of BBR intake can only affect lymphocyte proliferation and delayed-type hypersensitivity reaction, while 10 mg/kg of BBR suppresses both cellular and humoral immunity functions [108].

In another study, IP administration of BBR at a dosage of 5 mg/kg/day for 15 weeks caused atherosclerosis in *ApoE*−/− mice [109]. Regarding sub-chronic toxicity, BBR increases ALT and AST liver enzymes and damages the liver and lungs [110]. Another investigation revealed that, in diabetic rats, after 16 weeks of BBR intake at concentrations of >50, 100, and 150 mg/kg, liver tissue damage occurred, but these symptoms were not present in healthy rats [111]. Furthermore, exposure to BBR triggers uterine contraction and is also likely to generate teratogenic effects [112].

Treatment of PC12 cells with 10 and 30 μM BBR increased cytotoxicity, as indicated by increased apoptotic cell death. In vivo (5 and 30 mg/kg of BBR, IP administration for 21 days) and in vitro (10 and 30 μM BBR, for up to 48 h) studies on BBR against 6-hydroxydopamine (6-OHDA)-induced neurotoxicity in rats and PC-12 cells, respectively, presented a decrease in dopamine biosynthesis accompanied by lowered levels of norepinephrine [113]. Docking research demonstrated that BBR inhibited AChE, BChE, MAO-A, and MAO-B, and its LD_50_ values were 0.44, 3.44, 126, and 98.2 μM, respectively [114].

In a study on *Aedes atropatpus* mosquito larvae, the effects of BBR showed persistent toxicity and dramatically amplified cumulative mortality [115]. Furthermore, the acute toxicity of BBR on the free-living stages of *Ichthyophthirius multifiliis* (a pathogenic parasite that attacks goldfish), specifically theronts and tomonts, was evaluated. The results revealed that 15 mg/L of BBR could eliminate 99.30% of *I. multifiliis* theronts in 4 h as well as cause morphological changes in protomonts and reduce the number of ribosomes. The BBR LC_50_ for goldfish was 528.44 mg/L at 96 h, which is almost 67 times greater than the EC_50_ for killing theronts (7.86 mg/L). It can be inferred that BBR is an effective and safe potential pesticide for the eradication of *I. multifiliis* [116].

## 4. Conclusions

In this review, several studies on the use of BBR in diabetes and related issues were examined. BBR is an isoquinoline alkaloid with anticancer, anti-inflammatory, antioxidant, and antimicrobial properties. It has proven beneficial in the treatment of diabetes and its complications, such as neuropathy, nephropathy, retinopathy, cardiomyopathy, osteoporosis, hepatic damage, endothelial dysfunction, and vascular problems. Most of the research concerns the effect of BBR on insulin resistance and secretion. BBR can increase insulin resistance and reduce its secretion by enhancing the expression of insulin receptors, Akt, and AMPK and reducing NF-κB. Since inflammation is a cause of type 2 diabetes (T2D), BBR can stop the development of T2D due to its anti-inflammatory properties. The use of BBR can positively influence diabetes-induced arrhythmia by shortening the prolonged QTc interval and restoring the diminished K^+^ current and L-type Ca^2+^ current to their normal states.

**Figure 1:**
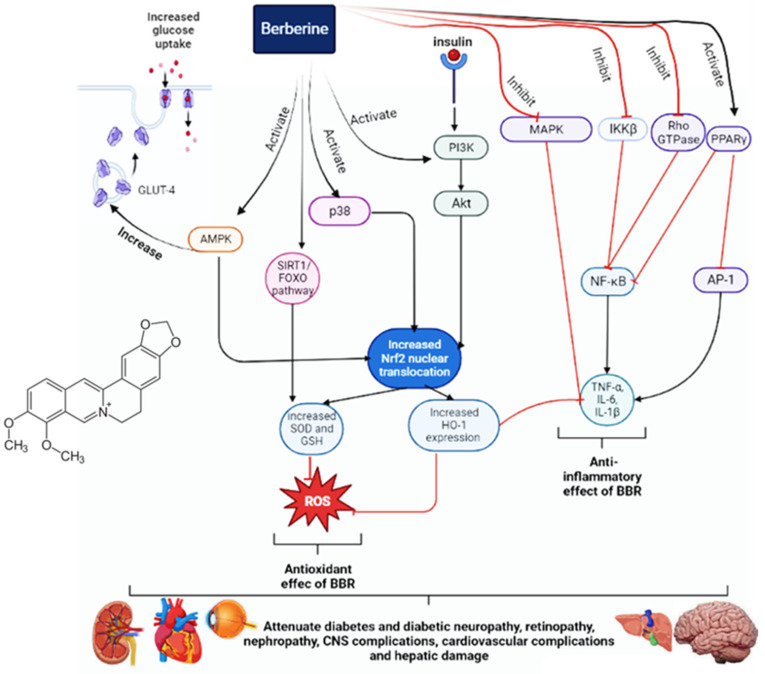
The antidiabetic mechanisms of berberine.

Furthermore, it can also reduce diabetes-induced fibrosis and cardiac dysfunction. BBR has beneficial effects for diabetic patients with hypertension due to its influence on vascular relaxation. It can also decrease the activities of AChE, BChE, and MAO, thereby improving cognitive performance, learning, and memory in diabetic patients. Furthermore, it can inhibit neuronal apoptosis and improve diabetic retinopathy by reducing VEGF levels. It can reduce hepatic gluconeogenesis and disorders of lipid metabolism, as well as provide a protective effect on the liver. The main antidiabetic mechanisms and effects of BBR are illustrated in Figure 1.

Regarding the toxicity of BBR, this depends on the amount present in a compound, the route of administration, and the type of organism; however, it is only moderately toxic in dogs. Currently, there is a lack of clinical trials to confirm the antidiabetic properties of BBR; therefore, further clinical studies are needed to support these claims.

## Figures and Tables

**Table 1:** Protective effects of berberine against cell models of DM.

Type of Extract or Constituent	Cell Line	Results	Ref.
BBR	3T3-L1 adipocytes and L6 myoblasts	Inhibited triglyceride accumulation in fully differentiated and undifferentiated adipocytes ↓ Adipogenic gene expression and levels of most lipogenic transcripts ↓ PPARs, CCAAT/enhancer binding proteins (C/EBPs), 11beta-hydroxysteroid dehydrogenase 1 (11β-HSD1), and aP2 ↑ AMPK and ACC in both adipocytes and myoblasts ↑ GLUT4 translocation in myoblasts	[12]
BBR	Neonatal rat cardiomyocytes exposed to hypoxia/reoxygenation with elevated glucose levels	↓ Myocardial cell death ↑ Bcl-2/Bax ratio ↓caspase-3 Activated phosphoinositide 3-kinase (PI_3_K)–Akt and AMPK and eNOS phosphorylation	[13]
BBR with Metformin	High-glucose-induced HepG2 cell line	↓ Total lipid content and triglyceride synergistic effects ↓ FAS and SREBP-1c	[14]
BBR	High-glucose-induced H9C2 cell	Reduced H9C2 cell line hypertrophy Promoted mitogenesis and destroyed damaged mitochondria Restored autophagic flux in damaged cardiomyocytes ↑AMPK	[15]

**Table 2:** Protective effects of berberine in animal models of DM.

Type of Extract or Constituent	Dose/Concentration	Study Model	Results	Ref.
Berberine chloride	50 mg/kg/day; orally for 45 days	STZ-induced diabetic rats	↓ Blood glucose and HbAlc ↑ Plasma insulin, hemoglobin, and body weight ↑ Pancreatic levels of SOD, CAT, GPx, GSH, vitamin E, and vitamin C ↓ LOOH and TBARS ↓ TNF-a, NF-kB, phospho-NF-kB-p65, COX-2, and iNOS ↓ Caspase-8, t-Bid, Bax, cytochrome-c, and cleaved caspase-3 ↑ Bcl-2 ↑ Anti-inflammatory mediator IL-10 and GLUT-2	[16]
	187.5 and 562.5 mg/kg; orally for 4 weeks	STZ-induced DM in rats	↓ FBG, TGs, TC, FFAs, and apolipoprotein B ↑ HDL and apolipoprotein AI	[17]
	100 mg/Kg per day; intragastrically for 6 weeks 10 mg/Kg/d; intraperitoneally for 4 weeks	STZ-induced DM in mice	↓ FINS, HOMA-IR, and FPG, and expression of TLR4, TNF-α, IL-1β and IL-6 ↓ Pathological damage and macrophage (MΦ) infiltration in pancreatic islets of diabetic mice Regulated the probiotics in the intestinal tract Blocked the nuclear translocation of NF-κB in THP-1-derived MΦs	[18]
	156 mg/kg per day; intragastrically for 12 weeks	STZ-induced DM in rats	↓ FINS, HOMA-IR, hyperlipidemia ↑ p-TORC2 levels Up-regulated expression of liver kinase B1, AMPK, and phosphorylated AMPK Down-regulated expression of the key gluconeogenic enzymes Inhibited TORC2 nuclear translocation in the liver tissues	[19]
	Diabetic rats: 75 and 150 mg/kg/day; orally twice a day for 15 days Diabetic mice: 200 mg/kg/day; orally for 3 weeks	STZ-induced DM in rats and KK-Ay diabetic mice	↓ FBG and FINS ↑ Expression of insulin receptor mRNA and PKC	[20]
	150 mg/kg/d; orally for 9 weeks	STZ-induced T2D hamsters	↑ Expression of LXRs and PPARs ↓ Expression of SREBPs in visceral white adipose tissue ↓ Body weight, total visceral white adipose tissue weight, blood glucose, FFAs, TC, LDL-c, and TGs ↑ Serum adiponectin ↓ Serum leptin, TNF-a, IL-6, and HOMA-IR ↓ Adipocyte size	[21]
	100 mg/kg/d; orally for 7 weeks	STZ-induced diabetic rats	↓ FBG, plasma-free fatty acids, CRP, TGs, and TC Improved glucose tolerance Inhibited DPP-4 and PTP-1B activities Moderately improved glucose homeostasis	[22]
	5 mg/kg/day; intraperitoneally for 3 weeks	ob/ob and STZ-induced diabetic mice	Improved insulin, glucose tolerance, and glucose metabolism ↓ Blood glucose levels, cAMP, hepatic gluconeogenesis, and gluconeogenic gene expression Suppressed glucagon-induced CREB phosphorylation	[23]
	5 mg/kg/day; intraperitoneally for 3 weeks	*db/db* mice	↓ Body weight ↓ Fat mass and the size of fat cells Food intake did not change Improved glucose tolerance	[12]
	100 mg/kg/d; orally for 2 weeks	*db/db* mice	Improved insulin resistance ↓ FBG Suppressed protein tyrosine phosphatase 1B ↑ Phosphorylation of insulin receptor, insulin receptor substrate1, and Akt	[24]
	Berberine 100mg/kg/d and Berberine 100 mg/kg/d+ stachyose 200 mg/kg/d; orally for 55 days	*db/db* mice	Improved glucose metabolism, the balance of α- and β-cells, and mucin-2 expression Increased abundance of Akkermansia muciniphila ↑ Fumaric acid level ↓ Metabolite all-transheptaprenyl diphosphate	[25]
	300 mg/kg/day; orally for 12 weeks	Alloxan-induced diabetic mice with renal injury	↓ NF-κB, and the ↑ IκB-α ↓ Levels of fibronectin, transforming growth factor-beta 1, and intercellular adhesion molecule-1	[26]
	380 mg/day; orally for 2 weeks	HFD-fed rats	↓ Body weight, plasma triglycerides, and insulin resistance	[12]
Dihydroberberine	100 mg/kg/day; orally for 2 weeks	HFD-induced insulin resistance in mice and rat	Improved effectiveness of BBR Better oral bioavailability than BBR ↓ Augmented adiposity, TGs, and insulin resistance	[27]
	5, 10 mg/kg/day; intraperitoneal injections for 4 weeks	HFD-fed mice	↓ Insulin resistance, body weight, and HOMA-IR ↑ Synthesis of liver glycogen and SIRT1 expression Regulated SIRT1/FOXO1 pathway	[28]
	100 mg/kg/day; orally for 4 weeks	Mitochondria isolated from the liver of HFD-fed rats	↑ Mitochondrial SirT3 activity Improved mitochondrial function Prevented a state of energetic deficit	[29]
Berberine	RB 0.7 (RB-L), 2.11 (RB-M), or 6.33 mg/kg/day (RB-H); orally for 8 weeks	High-sugar, high-fat diet (HSHFD)-induced diabetic KKAy mice	Improved glucolipid metabolism, insulin resistance, OGTT, insulin tolerance test (ITT), and pathological changes in the pancreases and livers of mice ↓ FBG, white fat index, TGs, LDL, GIP, and insulin level ↑ GLP-1, HDL, and glycogen content in the liver and muscle ↑ p-PI3K and p-AKT levels ↓ TXNIP expression	[30]
	150 and 300 mg/kg/day; gavage for 12 weeks	HFD-fed rats	↓ Body weight, urine volume, FBG, BUN, cholesterol, hepatic index levels, pathologic changes, and IR Improved albumin levels, glucose consumption, uptake, and inflammation ↑ Expression of PPM1B, PPARγ, LRP1, GLUT4, IRS-1, IRS-2, PI3K, AKT, and IKKβ Inhibited the phosphorylation of pIKKβ Ser181, total IKKβ, NF-κB p65, and JNK	[31]
	Berberine 100 mg/kg/d for 30 days then 150 mg/kg/d; berberine combined with stachyose; BBR 100 mg/kg/d + stachyose 200 mg/kg/d for 30 days then BBR 150 mg/kg/d + 300 mg/kg/d; 69 days in total	Zucker diabetic fatty rats	↓ Blood glucose Improved impaired glucose tolerance ↑ Abundance of beneficial Akkermansiaceae, ↓ Abundance of pathogenic Enterobacteriaceae, Desulfovibrionaceae, and Proteobacteria ↓ Expression of intestinal Egr1 and Hbegf ↑ Expression of miR-10a-5p (just combination therapy)	[32]

**Table 3:** Protective effects of berberine against DM complications (in vivo studies).

Constituent	Dose	Study Model	Results	Ref.
BBR	100 mg/kg; orally for 7 days	STZ-induced ischemic arrhythmias	Shortened prolonged QTc interval Returned the diminished K^+^ current and L-type Ca^2+^ current to their normal states	[47]
BBR	60 mg/kg/day; intragastrically for 14 days	STZ-induced ischemic arrhythmias	Increased K^+^ current and current density Increased Kir2	[10]
BBR	160 mg/kg/day; orally for 12 weeks	Lean and GDM-exposed mice offspring	↑ Cardiolipin remodeling enzyme tafazzin, tetra linoleoyl-cardiolipin, total cardiac cardiolipin ↓ NEFA, TGs, and ketones	[48]
Berberine chloride hydrate	0.5 g/L; added to drinking water for 14 weeks	Apoe^−/−^ HFD-fed mice	↓ Akkermansia spp. and Bacteroides ↓ TNF-α and IL-1β Increased colonic mucus layer thickness ↓ TC, LPS, VCAM-1, and MMP-2 ↑ ZO-1 and Occludin in the ileum and colon, respectively	[49]
BBR	50 mg/kg twice weekly; intragastrically for 12 weeks	BBR-treated Apoe^−/−^ HFD-fed mice cohoused with non-BBR-treated Apoe^−/−^ HFD-fed mice	↓ FMO3 and TMAO Changed the abundance of Firmicutes and Verrucomicrobia	[50]
BBR	200 mg/kg/day; orally for 4 weeks	STZ-induced cardiac dysfunction	Ameliorated cardiac fibrosis and dysfunction ↓ IGF-1R, MMP-2/MMP-9, alpha-smooth muscle actin, and collagen type I	[51]
BBR	50, 100, and 200 mg/kg/day; intragastrically for 8 weeks	STZ-induced hypertension	↓ Serum glucose and blood pressure Improved vascular relaxation Up-regulated expression of BKca	[52]
BBR	50, 100 mg/kg; orally for 6 weeks	High-fat diet-induced diabetic hamsters	Reduced susceptibility to cardiovascular complications of diabetes ↓ Body weight, insulin, and glucose level Inhibited hepatic fat accumulation Increased glucose tolerance	[53]
BBR	187.5 mg/Kg/d; intragastrically	STZ-induced cognitive decline	Down-regulated PI3K/Akt/mTOR and MAPK signaling pathway ↓ PKCη, PKCε, translocation of NF-κB, amyloid precursor protein, BACE-1, and Aβ42 ↑ GLUT3 and glucose uptake in the brain	[9]
BBR	25–100 mg/kg; orally twice daily for 30 days	STZ-induced memory dysfunction	↓ Hyperglycemia, oxidative stress, and AChE activity Improved cognitive performance, learning, and memory	[54]
BBR	50 and 100 mg/kg; orally for 14 days	STZ-induced impaired neurochemicals	Restore impaired neurochemicals ↓ AChE, BChE, MAO activities, and MDA ↑ SOD, GPx activities, and GSH	[55]
BBR	5, 10, and 20 mg/kg; intraperitoneally, single and repeated treatment (twice daily for 14 days)	STZ-induced neuropathy	↓ MDA, SOD, catalase, and GPx activities	[56]
BBR	50 mg/kg; orally for 10 weeks	*db/db* mice with encephalopathy	Improved learning and memory ability ↑ HDL, PSD95, SYN, NGF, and SIRT1 ↓ Body weight, FBG, TNF-α, NF-κB, TGs, TC, and LDL Down-regulated PERK, IRE-1α, eIF-2α, PDI, and CHOP	[57]
BBR	200 mg/kg/day; orally for 4 weeks	STZ-induced diabetic rats with cerebral ischemia/reperfusion injury	↑ Expression of PI3K, p-Akt, and Bcl-2 ↓ Cerebral infarct volume and cell apoptosis of cerebral infarct area ↓ NO and MDA ↓ Expression of Caspase-3 and Bax ↑ SOD	[58]
BBR	Dosage not mentioned; s.c. injection	STZ-induced neuropathy	Reduced neuropathy pain Suppressed the activation of microglia and astrocytes in the spinal cord ↓ Expression of TNF-α, IL-6, IL-1β, iNOS, and COX-2	[59]
BBR	50, 100, and 200 mg/kg/day; intragastrically for 8 weeks	STZ-induced vascular dysfunction	↓ FBG, the augmented contractile function of the cerebral artery to KCl and 5-HT, Ca^2+^ channel current densities, α1C-subunit expressions of Ca^2+^ channels, and resting intracellular Ca^2+^ level ↓ Ca^2+^ release from RyRs in cerebral VSMCs	[60]
BBR	187.75 mg/kg/day	STZ-induced cognitive impairment	Improved spatial learning memory Up-regulated α7nAchR expression ↓ AChE activity, inflammation, CSF/blood glucose, and Aβ	[61]
BBR	150 mg/kg; for 4 weeks	STZ-induced diabetic Alzheimer’s	Restored the disordered arrangement of nerve cells and damage to neurons ↓ GRP78, CHOP, procaspase-12, procaspase-9, and procaspase-3 in the hippocampus ↓ FBG, TGs, TC, glycosylated serum protein levels, Aβ, and apoptosis rate	[62]
BBR	5, 20, and 40 mg/kg/day; i.p. for 10 weeks	STZ-induced neuropathic pain	Increased mechanical and thermal nociception threshold ↓ ROS and MDA ↑ Catalase activity ↓ TNF-α and IL-6 Up-regulated expression of MOR	[63]
BBR	BBR 10 mg/kg+ gypenosides, 1 mg/kg+ bifendate 0.3 mg/kg; intragastrically for 14 weeks	*db/db* and STZ-induced diabetic mice	↓ FBG, body weight, TGs, LDL No positive effects on memory impairment Synergistic effect	[64]
BBR	10, 20, and 40 mg/kg; PO for 8 weeks	STZ-induced painful diabetic peripheral polyneuropathy	↓ FBG, food intake, water intake, urine output, hepatic cholesterol, TGs, MDA, NO, glycosuria, aldose reductase, glycated Hb, oxide-nitrosative stress and pulse Ox levels, TNF-α, IL-1β, and IL-6 ↑ Body weight, serum insulin, pulse Ox, SOD, GSH, thermal hyperalgesia, motor nerve conduction velocity (MNCV), sensory nerve conduction velocity (SNCV), BDNF, IGF-1, and PPAR-γ ↑ Thr-172 expression ↓ PP2C-α expression ↓ Necrosis, edema, infiltration of inflammatory cells, congestion in the sciatic nerve, and atrophy in myelinated axons	[65]
BBR	50 and 100 mg/kg; orally 0.2 and 0.4 μg/kg; ocular delivery for 12 weeks	Type 1 (STZ-induced) and type 2 (*db/db*) diabetic retinopathy mice treated with insulin	↓ VEGF and HIF-1α Inhibited the Akt/mTOR pathway in insulin-treated retina endothelial cells Inhibited progression of retinopathy in types I and II diabetes	[8]
BBR	In vivo: 40, 160 mg/kg; orally for 4 weeks 2	STZ-induced hepatic damage	↓ TC, TGs, LDL, AST, ALT, FBG, ISI, HNF-4α, miR122, PEPCK, G6Pase, FAS-1, and ACCα ↑ HDL, FINS, and CPT1 Attenuated hepatic gluconeogenesis and lipid metabolism disorder	[66]
BBR	In vivo: 200 mg/kg/day; gavage for 4 weeks Ex vivo: 2.5–10 μM	STZ-induced endothelial dysfunction	Improved mesenteric arteries’ insulin sensitivity Improved endothelium-mediated vasodilatation Up-regulated insulin receptor-mediated signaling Synergistic effects between insulin and berberine ↑ Phosphorylation of InsR, AMPK, Akt, and eNOS	[67]
BBR	200 mg/kg/day; for 10 weeks	HFD-fed mice	Improved insulin resistance Reduced the abundance of the bacteria that produce BCAAs Activated BCKDC ↓ Phosphorylation state of BCKDHA and BCKDK in the liver and epididymal white adipose tissues	[46]
BBR	100 and 200 mg/kg	STZ-induced diabetic nephropathy hamster	↓ Blood glucose, blood lipids, IL-1β, IL-6, NLRP3, Caspase-1, GSDMD, MDA, and the number of TUNEL-positive cells ↑ Nrf2 expression Improved NLRP3-Caspase-1-GSDMD signaling Inhibited diabetic nephropathic damage	[68]
BBR	150 mg/kg/d orally; for 12 weeks	STZ-induced diabetic kidney disease	↓ Microalbumin and renal pathologic changes and EMT Down-regulated NLRP3	[69]
BBR	200 mg/kg/day; for 8 weeks	STZ-induced diabetic nephropathy mice	Attenuated diabetic nephropathy Activated AMPK signaling pathway	[70]
BBR	200 mg/kg/day; intragastrically for 12 weeks	Diabetic rat kidneys	Suppressed RhoA/ROCK signaling ↓ NF-κB ↓ Intercellular adhesion molecule-1, transforming growth factor-beta 1, and fibronectin	[71]
BBR	50, 100, and 150 mg/kg; orally for 14 days	STZ-induced renal ischemic injury	↓ BUN, creatinine, and LDH ↑ Ca^2+^-ATPase and Na^+^/K^+^-ATPase enzyme activities Antioxidant, anti-inflammatory, and antiapoptotic effects	[72]
Berberine hydrochloride	100 mg/kg/d; gavage for 3 weeks	Zucker diabetic fatty rats	↓ Food intake, FBG, insulin resistance, and LPS ↑ Fasting GLP-2, glutamine-induced intestinal GLP-2 secretion, goblet cell number, and villi length ↑ Mucin, occludin, and ZO-1 ↓ TLR-4, NF-κB, and TNF-α	[73]
BBR	200 mg/kg/day; intragastrically for 6 weeks	STZ-induced diabetic rats	↑ Bacteroidetes and Lactobacillaceae ↓ Proteobacteria and Verrucomicrobia ↓ Aromatic amino acids, such as tyrosine, tryptophan, and phenylalanine	[74]
BBR	120 mg/kg/day; orally for 4 weeks	STZ-induced osteoporosis	Improved glucose and bone metabolism	[75]
BBR	BBR (210 mg/kg) BBR (210 mg/kg) + oryzanol (33.6 mg/kg) + vitamin B6 (7 mg/kg); orally (1 mL/100 g body weight) for 4 weeks	Diabetes-induced gut microbiota alteration *db/db* mice	↓ FBG, HbA1c ↑ Bacteroidaceae and Clostridiaceae ↑ DCA, TGR5, GLP, and glucose, lipid, and energy metabolism	[76]

**Table 4:** Protective effects of berberine against DM complications (in vitro studies).

Study Model	Results	Ref.
H9C2 cell line	↓ High-glucose-induced hypertrophy Improved mitochondrial function Promoted mitogenesis Activated AMPK signaling Restored autophagic flux	[15]
AML12 hepatocytes and 3T3-L1 adipocytes	↓ BCAAs	[46]
High-glucose-induced BMSCs cell line	Increased osteogenesis Up-regulated ROS-mediated IRS-1 signaling pathway	[75]
Palmitate-incubated HepG2 cells	↓ HNF-4α, miR122, PEPCK, G6Pase, FAS-1, and ACCα ↑ CPT1	[66]
High-glucose-induced rat retinal Müller cells	Reduced apoptosis Increased autophagy ↓ Expression of Bax and caspase-3 ↑ Expression of Bcl-2 ↑ Beclin-1 and LC3II ↑ AMPK/mTOR signaling pathway	[77]
In vitro model of high-glucose-AGE-induced micro-endothelial injuries	↑ Thrombomodulin, NOS, and NO Inhibited AGEs formation	[78]
High-glucose-induced endothelial dysfunction in endothelial cells and blood vessels isolated from rat aorta	↑ eNOS and NO ↓ ROS, cellular apoptosis, NF-κB, and expression of adhesion molecules Inhibited monocyte attachment to endothelial cells Increased endothelium-dependent vasodilatation Activated AMPK	[79]
Palmitate-induced endothelial dysfunction in human umbilical vein endothelial cells (HUVECs)	↑ Expression of eNOS ↓ Expression of NOX4 ↑ Expression of AMPK and p-AMPK ↑ NO ↓ ROS	[80]
High-glucose-induced SH-SY5Y human neuroblastoma cells	↑ Nrf2, HO-1 and NGF Inhibited neuronal apoptosis ↓ Cytochrome c and ROS ↑ Bcl-2 expression and IGF-1/Akt/GSK-3β signaling pathway	[81]
High-glucose-induced HK-2 cells	↓ EMT Down-regulated NLRP3	[69]
Palmitate-induced lipid accumulation and apoptosis in HK-2 cells	↑ CPT1A, PPARα, and PGC1α Reversed intracellular lipid accumulation and apoptosis Promoted fatty acid oxidation	[82]
Palmitic acid-induced cultured podocyte	Improved podocyte damage Inhibited lipid accumulation, excessive production of mitochondrial ROS, mitochondrial dysfunction, and deficient fatty acid oxidation Restored PGC-1α	[83]
High-glucose-induced renal fibrosis	Suppressed RhoA/ROCK signaling ↓ NF-κB ↓ Fibronectin overexpression ↓ Excessive reactive oxygens	[71]
High-glucose-induced renal fibrosis	Activated TGR5 Inhibited S1P2/MAPK signaling ↓ Fibronectin, ICAM-1, and TGF-β1 Down-regulated phosphorylation level of c-Jun/c-Fos	[84]
Insulin-resistant rat H9c2 cardiomyocyte	Reduced insulin resistance Increased glucose consumption and glucose uptake Activated AMPK	[85]
Palmitate-induced insulin-resistant H9c2 cardiomyocytes	↑ Glucose uptake and consumption Activated AKT ↑ GLUT-4 ↓ DAG and TAG hydrolysis ↑ TAG and expression of DAG acyltransferase-2 Increased palmitic acid, [1,3-3H] glycerol, and [1-14C] glucose incorporation into TAG and decreased their incorporation into DAG	[86]
Insulin-treated human sebocytes (SEB-1)	Inhibited sebocyte apoptosis Reduced susceptibility to cardiovascular complications of diabetes ↓ Expression of BIK protein	[53]

## Data Availability

No data were used to support this study.

## Abbreviations

Akt1: Protein kinase B; ALA: Alpha-linolenic acid; ALT: Alanine transaminase; AMPK: Activation of 5′ AMP-activated protein kinase; AR: Androgen receptor; AST: Aspartate transaminasel; Aβ: β-Amyloid; BCAA: Branched-chain amino acid; BDNF: Brain-derived neurotrophic factor; CaMKβ: Calmodulin-dependent protein kinase ꞵ; CRF: Cardiac risk factor; CREB: Cyclic AMP response element binding; ErbB2: Erb-b2 receptor tyrosine kinase 2; ET-1: Endothelin-1; FAS: Fatty acid synthase; FBG: Fasting blood glucose; FFA: Free fatty acid; FINS: Fasting serum insulin; FPG: Fasting plasma glucose; FST: Forced swimming test; GLP1: Glucagon-like peptide-1; GLUT: Glucose transporter; GSH: Glutathione; GSK: Glycogen synthase kinase; HbA1c: Hemoglobin A1C; HDL: High-density lipoprotein cholesterol; HOMA-IR: Homeostatic model of assessment of insulin resistance; ICAM-1: Intercellular adhesion molecule-1; IFN-γ: Interferon-gamma; IGF: Insulin-like growth factor; IL-6: Interleukin-6; ISI: Insulin sensitivity index; IκBα: Inhibitor of NF-κB; LDL: Low-density lipoprotein cholesterol; LTB4: Leukotriene B4; MAO: Monoamine oxidase; MDA: Malondialdehyde; MMP: Matrix metalloproteinase; MWM: Morris water maze; MΦ: Macrophage; NF-κB: Nuclear factor kappa-B; OGTT: Oral glucose tolerance test; OVX: Ovariectomized; PBG: Postprandial blood glucose; PI3K: Phosphatidylinositol-3 kinase; PPAR: Peroxisome Proliferator-activated receptor; QUICKI: Quantitative insulin sensitivity check index; ROCK: Rho-associated protein kinase; SNP: Sodium nitroprusside; SOD: Superoxide dismutase; SREBP-1c: Sterol regulatory element-binding protein 1c; STZ: Streptozotocin; T2D: Type 2 diabetes; TAS: Total antioxidant status; TBAR: Thiobarbituric acid reactive substance; TC: Total cholesterol; TG: Triglyceride; TGF-β1: Transforming growth factor-β1; TNF-α: Tumor necrosis factor-α; TPS: Tail pinch stressor; VCAM-1: Vascular cell adhesion molecule-1; VEGF: Vascular endothelial growth factor; VGSC: Voltage-gated Na+ channel.
